# Plant-Growth Promoting Microbes Change the Photosynthetic Response to Light Quality in Spinach

**DOI:** 10.3390/plants12051149

**Published:** 2023-03-03

**Authors:** Luca Vitale, Ermenegilda Vitale, Silvana Francesca, Christian Lorenz, Carmen Arena

**Affiliations:** 1 National Research Council, Department of Biology, Agriculture and Food Sciences, Institute for Agricultural and Forestry Systems in the Mediterranean, P. le E. Fermi 1, 80055 Portici, Italy; 2 Department of Biology, University of Naples Federico II, Via Cinthia, 80126 Napoli, Italy; 3 Department of Agricultural Sciences, University of Naples Federico II, Via Università 100, 80055 Portici, Italy; 4 NBFC-National Biodiversity Future Center, 90133 Palermo, Italy

**Keywords:** light quality, plant growth promoting microorganisms, productivity, chlorophyll a fluorescence, ribulose-1,5-bisphosphate carboxylase-oxygenase, *Spinacia oleracea* L., photosynthetic plasticity

## Abstract

In this study, the combined effect of plant growth under different light quality and the application of plant-growth-promoting microbes (PGPM) was considered on spinach (*Spinacia oleracea* L.) to assess the influence of these factors on the photosynthetic performance. To pursue this goal, spinach plants were grown in a growth chamber at two different light quality regimes, full-spectrum white light (W) and red-blue light (RB), with (I) or without (NI) PGPM-based inoculants. Photosynthesis-light response curves (LRC) and photosynthesis-CO_2_ response curves (CRC) were performed for the four growth conditions (W-NI, RB-NI, W-I, and RB-I). At each step of LRC and CRC, net photosynthesis (*P*_N_), stomatal conductance (*g*_s_), *C*_i_/*C*_a_ ratio, water use efficiency (WUE_i_), and fluorescence indexes were calculated. Moreover, parameters derived from the fitting of LRC, such as light-saturated net photosynthesis (*P*_Nmax_), apparent light efficiency (Q_pp_), and dark respiration (*R*_d_), as well as the Rubisco large subunit amount, were also determined. In not-inoculated plants, the growth under RB- regime improved *P_N_* compared to W-light because it increased stomatal conductance and favored the Rubisco synthesis. Furthermore, the RB regime also stimulates the processes of light conversion into chemical energy through chloroplasts, as indicated by the higher values of Q_pp_ and *P*_Nmax_ in RB compared to W plants. On the contrary, in inoculated plants, the *P_N_* enhancement was significantly higher in W (30%) than in RB plants (17%), which showed the highest Rubisco content among all treatments. Our results indicate that the plant-growth-promoting microbes alter the photosynthetic response to light quality. This issue must be considered when PGPMs are used to improve plant growth performance in a controlled environment using artificial lighting.

## 1. Introduction

The manipulation of spectral light composition was successfully used to optimize the growth of plants [1] and is currently applied in horticulture to improve the yield and quality of many crops, as it triggers a wide range of changes to primary and secondary plant metabolism [2,3]. Different approaches were used to modify the spectral quality, such as the pho-to-selective nets or films [4,5], colored shade nets [6], and, recently, the artificial lighting systems [7].

Light-emitting diode (LED) technology can optimize the ratios between the various wavelengths useful for the plant and offer numerous advantages over traditional forms of lighting, such as high luminous efficiency, reduced energy consumption and costs, and low heat production. In addition, LED utilization also improved the synthesis of bioactive compounds in plant tissues beneficial for human health [8].

Light quality (LQ) and different wavelengths were reported to affect plant photosynthesis and, in turn, the plant carbon gain. Plants use mainly the red and blue wavelengths in the photosynthetic process. Red light, absorbed by proper receptors (i.e., phytochromes), is known to play a role in controlling the functions of the chloroplast, photosynthetic apparatus development, stem and petiole growth, and the reproductive system; the blue light, through the blue light receptors (i.e., cryptochromes and phototropins), affects stomatal opening, photosynthesis, Rubisco and pigment synthesis, biomass production, as well as photomorphogenesis [2,9]. Although red and blue wavelengths efficiently regulate leaf photosynthesis, the contribution of green light cannot be neglected, as it increases photosynthetic efficiency in the inner layers of photosynthetic parenchyma [10]. Recently, Xie et al. [11] demonstrated that combining red–blue–green wavelengths enhances photosynthesis in *Bidens pilosa* L. plants compared to red-blue light. Based on these results, an inadequate light environment for plants limits photosynthesis.

In addition to the light growth environment, soil fertility also pushes crop production. In this framework, the plant growth-promoting microbes (PGPM)-based inoculants represent an eco-friendly and helpful technique for improving crop productivity and food quality in indoor and open-field cultivations [12]. Furthermore, Rhizosphere bacteria and mycorrhizal fungi can colonize plant roots providing benefits to their hosts by improving the availability of soil nutrients and the resistance against pathogens [13,14] and abiotic stress [15]. Conversely, the host plant sustains symbiotic costs by supplying photosynthates for microbe metabolism and growth. PGPM also act as a biofertilizer by promoting plant growth, acting on phytohormones metabolism [16]. This aspect is relevant since some phytohormones play a central role in regulating photosynthesis [17]. It is now known that beneficial soil bacteria produce phytohormones, among those the indole-3- acetic acid, IAA, and the abscisic acid, ABA, which are able to enhance the photosynthetic capacity in plants [18], while AMF-inoculated plants showed higher concentrations of plant hormones, including indole acetic acid, IAA, indole butyric acid, IBA, gibberellic acid, GA, and abscisic acid, ABA [19], which are known to have a benefit on plant photosynthesis.

Although several investigations were conducted on the impact of the light spectrum or PGPM on plant physiology, only a few studies were performed on the combined effects of LQ and PGPB on photosynthesis. Some of them focused on the role of light on phyllo-sphere microorganisms [20,21] or the role of light on the microbes of the rhizosphere in phytoremediation [11].

Recently, it has been demonstrated that interaction between light quality and L-amino-acids-based biostimulant application may consistently affect photosynthetic and biochemical traits in soybean plants [8]. Therefore, it is likely that any changes in plant growth light environment could alter the cost–benefits inside the host plant, modifying the symbiosis.

In the present study, we chose *Spinacia oleracea* L. because it is among the most consumed vegetable worldwide, and previous research showed an adaptation of spinach to different light spectrum and PGPM application [15,22]. Therefore, we analyzed the photosynthetic behavior of spinach plants inoculated with plant-growth-promoting microorganisms in response to changes in the light spectrum. Considering that both light quality and PGPM may exert potential beneficial effects on plant carbon metabolism, this study aimed to assess if the plant growth under specific light quality regimes, in combination with PGPM inoculation, may elicit synergic or antagonistic effects on the photosynthetic performance of spinach, since plants provide energy through photosynthates to sustain the interaction with beneficial soil microbes. To pursue this goal, we explored the photosynthetic mechanisms of *S. oleracea* plants treated with an inoculant containing a cocktail of arbuscular mycorrhizal fungi, saprophytic fungi, and rhizosphere bacteria, analyzing gas exchanges, photo-chemical indexes, and Rubisco content.

## 2. Results

### 2.1. Effect of Light Quality and PGPM Application on Spinach Growth

The effects of LQ and PGPM as independent factors and their interaction were evaluated on plant growth, as reported in Table 1.

**Figure 1:**
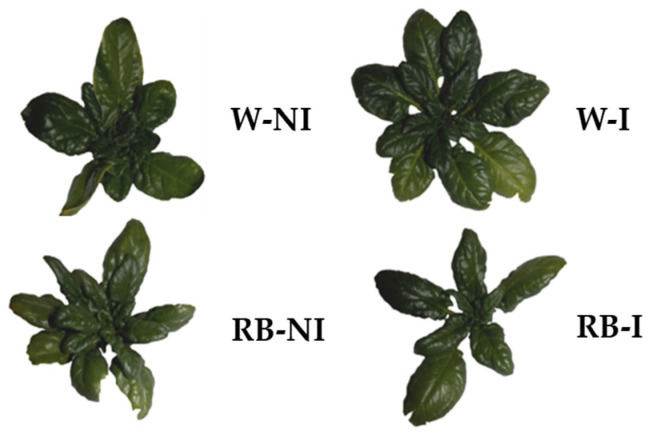
Representative images of not-inoculated (NI) and inoculated (I) spinach plants grown under W and RB light regimes.

LQ and PGPM and their interaction did not affect the leaf number. The total leaf area (Table 1, Figure 1) was significantly influenced by the factor LQ. It was higher (*p* < 0.01) in W plants than RB and reached the minimum value in RB-I plants.

Furthermore, LQ and PGPM treatments consistently affected the S/B ratio, which increased (*p* < 0.01) under the W-light compared to the RB-light and in NI plants compared to I plants. Among all treatments, W-NI plants exhibited the highest S/B ratio (*p* < 0.01). Finally, R/B was only affected by LQ, showing lower (*p* < 0.01) values in W than RB plants, regardless of PGPM application.

### 2.2. Effect of Light Quality and PGPM Application on Light Response Curve (LRC)

**Figure 2:**
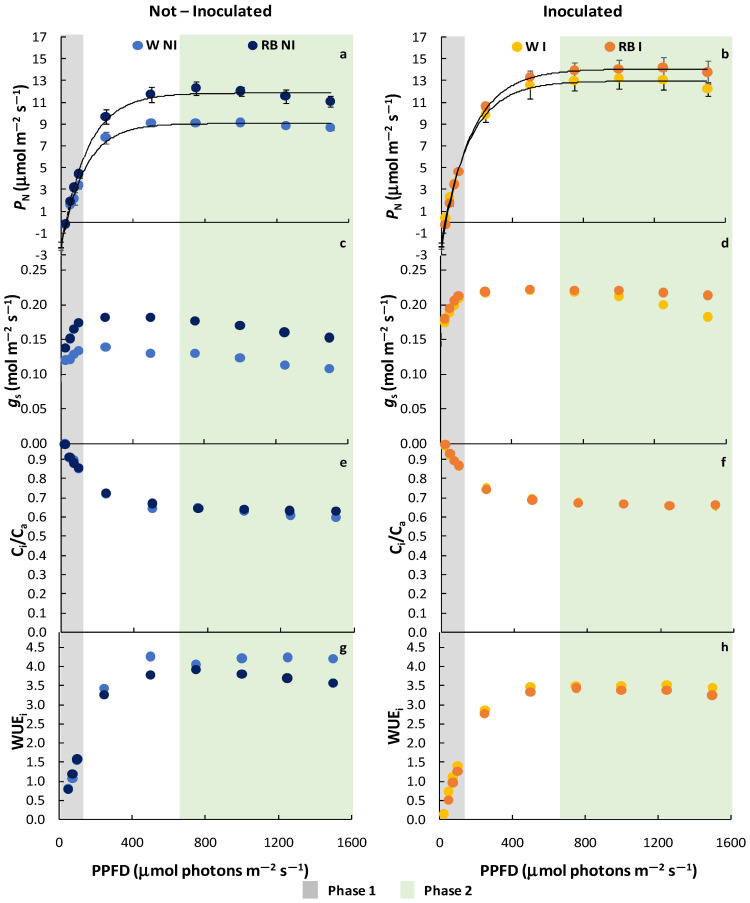
Net photosynthesis, *P*_N(LRC)_ (**a**,**b**); stomatal conductance, *g*_s_ (**c**,**d**); internal to atmospheric CO_2_ concentration ratio (*C*_i_/*C*_a_) (**e**,**f**); instantaneous water use efficiency, WUE_i_ (**g**,**h**) of not-inoculated (NI) and inoculated (I) spinach plants grown under W and RB light regimes. Data are means ± standard error (n = 5). phase 1 (grey) and phase 2 (green).

**Figure 3:**
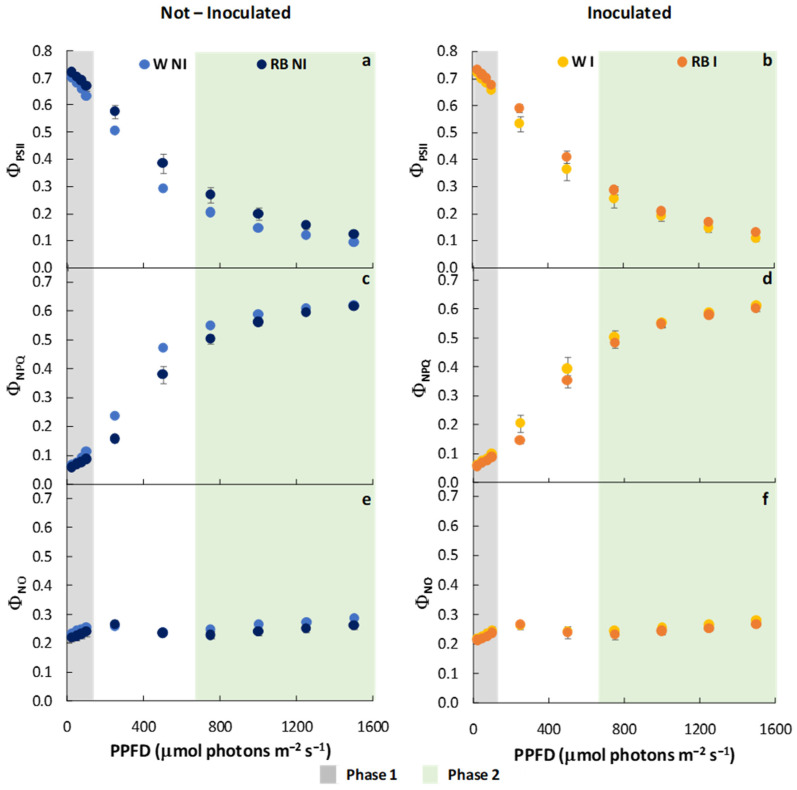
Effective quantum yield of PSII, Φ_PSII_ (**a**,**b**); regulated energy dissipation of PSII, Φ_NPQ_ (**c**,**d**); not-regulated energy dissipation of PSII, Φ_NO_ (**e**,**f**) of not-inoculated (NI) and inoculated (I) spinach plants grown under W and RB light regimes. Data are means ± standard error (n = 5). phase 1 (grey) and phase 2 (green).

The effect of LQ regimes and PGPM application as independent factors and their interaction on photosynthetic light response curve (LRC) are reported in Figure 2 and Figure 3 and in Table 2. In addition, a separate analysis was conducted on the entire curve, on the phase 1 and phase 2.

The analyses on the complete LRC revealed that *P_N_* (LRC) was influenced only by PGPM, showing higher (*p* < 0.05) values in I plants. No difference was observed in phase 1, except for the significant interaction LQ × PGPM. In this case, the *P_N_* of RB-I increased compared to W-I plants and even more than W-NI. Conversely, LQ, PGPM, and their interaction consistently influenced *P_N_*_(LRC)_ in phase 2 (Table 2), determining a better performance (*p* < 0.001) in plants grown under red-blue (RB) than under white (W) light and I than NI plants. Among all treatments, *P_N_*_(LRC)_ reached the highest value in RB-I plants (Figure 2a,b).

LQ, PGPM, and their interaction LQ × PGPM significantly affected stomatal conductance (Table 2). Plant growth under RB increased *g_s_* (*p* < 0.001) compared to the W regime; this result was strengthened in inoculated (I) plants, which exhibited higher values of *g*_s_ (*p* < 0.001) compared to non-inoculated (NI) ones. Interestingly, in phase 1, *g_s_* of both W-I and RB-I increased compared to NI plants, while in phase 2, an appreciable rise (*p* < 0.001) was evident only in RB-I plants (Figure 2c,d).

From the analysis of the entire curve, no difference was observed in *C_i_/C_a_* ratio and WUE_i_. In phase 1, PGPM as the main factor and its interaction with LQ significantly affect both *C_i_/C_a_* and WUE_i_. In phase 2, a significant effect was obtained only for the factor PGPM (Table 2). More specifically, I plants showed higher *C_i_/C*_a_ and lower WUE_i_ values (*p* < 0.001) than NI plants, regardless of the LQ regime (Figure 2e,f), with the highest values in W-NI plants (Figure 2g,h).

LQ, PGPM, and their interaction did not affect Φ_PSII_ and Φ_NPQ_ on the entire curve. However, a significant effect was induced by the interaction of LQ × PGPM in phase 1. Only PGPM, as a single factor, significantly influenced Φ_PSII_ and Φ_NPQ_ in phase 2 (Table 2). In particular, I showed higher Φ_PSII_ and lower Φ_NPQ_ values than NI plants, regardless of LQ regime (*p* < 0.05). Moreover, RB-NI plants were characterized by higher (*p* < 0.001) Φ_PSII_ compared to W-NI ones, while no difference occurred between W-I and RB-I plants (Figure 3a,b). Additionally, among all treatments, RB-NI maintained a lower (*p* < 0.01) Φ_NPQ_ than RB-I plants (Figure 3c,d).

LQ, PGPM, and their interaction (LQ × PGPM) significantly affected Φ_NO_, considering the entire curve and phase 2, while in phase 1, only LQ influenced Φ_NO_ (Table 2). Φ_NO_ was higher in W than in RB plants, regardless of the PGPM application (*p* < 0.01). Furthermore, an appreciable decrease in Φ_NO_ was observed in I compared to NI plants (Table 2, Figure 3e,f). Particularly in phase 2, W-NI exhibited higher (*p* < 0.01) values of Φ_NO_ compared to all other treatments.

### 2.3. Effect of Light Quality and PGPM Application on CO_2_ Response Curve (CRC)

**Figure 4:**
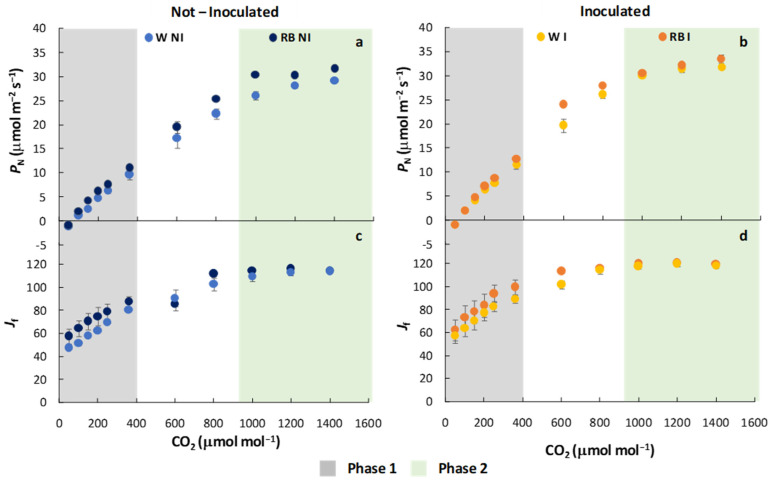
Net photosynthesis, *P*_N(CRC)_ (**a**,**b**); electron transport rate, *J*_f_ (**c**,**d**) of not-inoculated (NI) and inoculated (I) spinach plants grown under W and RB light regimes. Data are means ± standard error (n = 5). phase 1 (green) and phase 2 (grey).

LQ and PGPM as independent factors and their interaction were also evaluated on CO_2_-derived photosynthetic functional parameters, as shown in Table 3 and Figure 4. Similarly to the LRCs, separate analyses were run on the entire curve and on the phase 1 and the phase 2. The observations relative to the complete CRC curve showed that LQ, PGPM, and the interaction LQ × PGPM did not affect *P_N_*_(CRC)_. However, in phase 1, PGPM as the main factor and LQ × PGPM interaction significantly influenced *P_N_*_(CRC),_ while in phase 2, PGPM exerted a significant effect on photosynthesis (Table 3). In detail, *P_N_*_(CRC)_ was higher in I than in NI plants, irrespective of the LQ regime (*p* < 0.05), especially in phase 2. Furthermore, the highest *P_N_*_(CRC)_ was observed in RB-I, while the lowest in W-NI plants (Table 3 and Figure 4a,b).

The *J*_f_ parameter was significantly affected by LQ, PGPM, and their interaction along the entire curve and in phase 1. More specifically, RB exhibited a higher value (*p* < 0.01) of *J*_f_ compared to W plants in both NI and I plants; interestingly, as observed for P_N(CRC)_, the lowest *J*_f_ was observed in W-NI while the highest in RB-I plants (Table 3, Figure 4c,d). In phase 2, only PGPM influenced the *J*_f_ parameter, which showed higher *J*_f_ values in I (*p* < 0.001) than NI plants.

### 2.4. Effect of Light Quality and PGPM Application on SPAD and Photosynthetic Functional Traits of LRC

The effect of LQ and PGPM as independent factors and their interaction on SPAD and photosynthetic functional traits of spinach plants are shown in Table 4.

LQ, PGPM, and their interaction did not influence SPAD or *F*_v_/*F*_m_ ratio, while *R*_d_ was affected only by LQ, which determined a reduction in *R*_d_ of RB-I compared to W-I plants, respectively, from PGPM (Table 4). Conversely, the parameters *P*_Nmax_ and Q_pp_ were significantly affected by PGPM and LQ as independent factors and their interaction. More specifically, *P*_Nmax_ and Q_pp_ were significantly higher in RB than in W plants and in inoculated compared to not-inoculated plants. *P*_Nmax_ was enhanced by 30% in W-I plants compared to not-inoculated plants while in RB-I plants, *P*_Nmax_ was enhanced by 17%. Furthermore, the interaction LQ × PGPM evidenced the highest values for RB-I plants and for W-NI plants the lowest values of *P*_Nmax_ and Q_pp_, respectively (Table 4).

### 2.5. Effect of Light Quality and PGPM Application on Rubisco Protein

**Figure 5:**
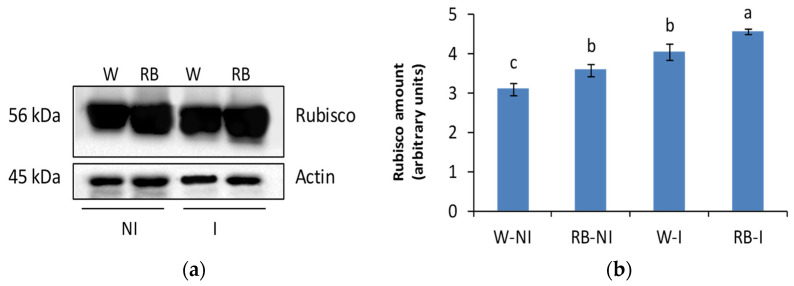
Western blot and densitometric analysis of Rubisco protein (**a**,**b**) in not-inoculated (NI) and inoculated (I) spinach plants grown under W and RB light regimes. Data are means ± standard error (n = 5). Different letters denote significant differences.

Rubisco amount was significantly affected by LQ (*p* < 0.01) and PGPM (*p* < 0.001). RB increased (*p* < 0.01) the Rubisco content compared to the W light regime. PGPM treatment induced a higher (*p* < 0.001) protein concentration in I- than NI-plants. All treatments determined a progressive rise of Rubisco content compared to W-NI plants, with the highest amount in RB-I plants (Figure 5a,b). Compared to W-NI, the Rubisco percentage increase was 16%, 33%, and 51% in RB-NI, W-I, and RB-I plants, respectively. Moreover, within the W light regime, I-plants exhibited a 30% increase in Rubisco than NI plants, while RB-I plants produced an increase in protein of 27% to RB-NI plants.

## 3. Discussion

### 3.1. Effect of Light Quality on Spinach Growth and Photosynthetic Performance

Light is the driving force for photosynthesis, but some wavelengths are preferentially used by the photosynthetic apparatus. Indeed, red and blue wavelengths are traditionally believed to have a higher quantum yield of photosynthesis than other visible wavelengths [23] and therefore, are often used in plant growth under artificial lighting.

In our study, the plant growth under red-blue (RB) regime enhanced photosynthesis compared to white (W) and this may depend both on the stimulation that blue wavelengths exert on stomatal opening (which, in turn, promotes a greater CO_2_ uptake) and on the stimulation of the Rubisco synthesis [24], which ensures a high uptake of intercellular CO_2_ in Calvin–Benson cycle. Moreover, our data showed that *P*_N(LRC)_ obtained by light response curves was significantly lower than *P*_N(CRC)_ derived from CO_2_ response curve and this result could be ascribed to both the stomatal and not-stomatal limitations of CO_2_ concentration at carboxylation sites. In both LRCs and CRCs, the beneficial effect of RB regime on photosynthesis of *S. oleracea* mainly occurred in the region where the carboxylation by Rubisco enzyme limited the CO_2_ assimilation. It was likely that the higher percentage of red and blue light of RB compared to full white regime promoted the light use efficiency in the chloroplast.

It is noteworthy that red-blue lights are both fundamental for photosynthesis process; however, the deficiency of blue light should compromise photosynthesis more than red deficiency [25]. Based on the existing literature, we attributed the better performance of photosynthetic apparatus observed under RB regime to a high blue amount which promoted a greater Rubisco synthesis in spinach chloroplasts and a better capability to use light under low light intensities (Q_pp_). Our results were consistent with a previous study of Li et al. [26], who demonstrated that RB-light promoted Q_pp_, leading to a rise in *P*_Nmax_. This outcome indicated that RB-light regime improves photosynthetic because it stimulates the processes of light conversion into chemical energy through chloroplasts, as indicated by the higher values of Q_pp_ and *P*_Nmax_ in RB compared to W plants. The growth under RB-light determines a reduced leaf area expansion and a greater carbon investment in root than shoot, highlighting the positive influence of blue light on root development and the requirement of blue wavelength for the optimal growth of spinach [8,22].

Under RB-light, Φ_PSII_ was upregulated in phase 2 of LRC as consequence of the enhanced CO_2_ assimilation related to dark-reactions of photosynthesis; this lead to a significant reduction of non-regulated energy (Φ_NO_) dissipation. A potential rise of Φ_NO_ highlights conditions enhancing the production of reactive oxygen species, dangerous for photosystem functionality, which generally occurs under high light.

### 3.2. Effect of PGPM on Plant Growth and Photosynthesis

The inoculation of crops with plant-growth promoting microbes is currently a good practice to improve plant yield and quality because beneficial microbes control the synthesis of plant growth-regulators, solubilize soil insoluble minerals, and increase the plant tolerance to biotic and abiotic stress [27]. According to our previous results [28], the treatment of spinach plants with inoculant containing a cocktail of arbuscular mycorrhizal fungi, saprophytic fungi, and rhizosphere bacteria (PGPM) modified the physiological mechanisms related to photosynthesis if compared to not-inoculated (control) plants. We argue that in W-I plants, the microbic symbiotic association improved photosynthesis, enhancing stomatal conductance and stimulating the Rubisco synthesis, with a direct consequence of more efficient CO_2_ harvesting from substomatal cavities and carboxylation. PGPM have likely promoted a higher water uptake, enhancing the plant water relationships, as well as the nitrogen uptake. We hypothesized that the nitrogen was mainly allocated towards Rubisco, as indicated by the higher enzyme content, whose activity is positively correlated with leaf nitrogen [29]. Our results also confirmed the positive role of PGPM on root development showing, in the W-I plants, a higher carbon investment in root compared to not-inoculated plants (W-NI).

### 3.3. Light and PGPM on Growth and Photosynthesis

A few studies explored the possible interactive effects between light quality and PGPM application on plant growth and, in particular, on photosynthetic performance. In our study, photosynthesis increased in inoculated compared to not-inoculated plants under both RB and W regimes; however, the plant growth under the full light spectrum showed the best result. However, even if PGPM promoted photosynthesis in RB-I by 17% compared to RB-NI plants, these benefits did not surpass those obtained growing inoculated plants under the W light, despite the increase of 27% of Rubisco content in RB-I. Our results seemed to suggest that PGPM effect on photosynthesis is antagonistic to the effect sorted by dichromatic RB light. It was demonstrated that the Rubisco activation state was reduced at high N leaves, which might limit the light-saturated CO_2_ assimilation [29], even if this did not occur in spinach plants [30]. Based on Evans and Takashima’s findings, we ascribed the response of RB-I plants to the lack of wavelengths critical for photosynthesis other than red and blue. It is well recognized that plants also need green wavelengths in photosynthesis, which, upon penetrating the deeper layers of the leaf more than red and blue light, excite the photosystems in inner cells of parenchyma [23]. It was likely that under RB-light, the energetic costs paid by plants for symbiosis with PGPM were excessive and may have determined a decrease in leaf photosynthesis and leaf area expansion, even if the plant had the need to invest heavily in Rubisco synthesis to sustain symbiosis. This hypothesis seems to be also endorsed by the highest respiratory rates (*R*_d_) in RB-I compared to W-I plants, which, on the contrary, reduced *R*_d_. It may be argued that plants–microbes interaction alters the photosynthetic response to light quality, and that the benefits of microbes occur only under a full light spectrum. This finding is of fundamental importance in the cultivation plannings under artificial lighting by using plant-growth promoting microbes.

Concerning the photosynthesis response curves, the plant–microbes interaction exerted beneficial effects both on phase 1 and phase 2 in both LRC and CRC, at least for W-I plants. Under RB light, the inoculum did not enhance the quantum yield of photosynthesis (Q_pp_), conversely to plants grown under full visible spectrum. Differently from not-inoculated plants, inoculated W plants exhibited a better capability to utilize light under low light intensities (Q_pp_) than RB-I plants. In this latter group, RB regime did not promote Q_pp_; so, we observed a small increase in *P*_Nmax_ compared to W plants. It is likely that under symbiosis, RB-light did not concur to improve photosynthetic capacity, as occurred under the full light spectrum, suggesting a limitation of RuBP regeneration in the carbon reduction cycle.

Inoculum improved the PSII quantum efficiency (Φ_PSII_) both in phase 1 and phase 2 of LRC and CRC, indicating that the PGPM determined a benefit also on the light use efficiency in photochemistry. However, our data demonstrated that the inoculum changed the energy partitioning within PSII. In particular, both in phase 1 and phase 2, RB-I plants invested more energy in photochemistry decreasing Φ_NPQ_, while in W-I plants, the higher photochemistry reduced the Φ_NO_. Our results indicated that RB inoculated plants dissipated the excess of light energy less efficiently than W inoculated plants because the reduction of non-photochemical mechanisms lead to a rise in Φ_NO_.

## 4. Material and Methods

### 4.1. Plant Growth Conditions

**Figure 6:**
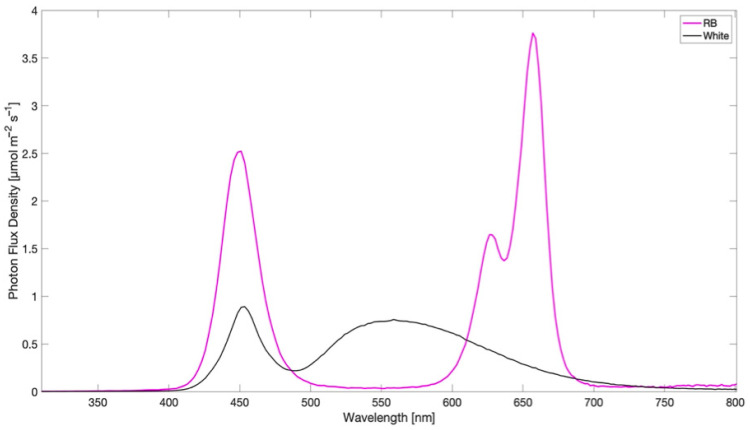
Light spectra set-up in the growth chamber: broad-spectrum white (W) and red-blue light (RB). Emission peaks for Red at 620 and 660 nm, emission peak for Blue at 460 nm).

Seeds of spinach (*Spinacia oleracea* L.) were sown in 0.5 L plastic pots filled with a mixture of sterilized sandy soil and perlite substrate (3:1, *v/v*) and were moved inside a home-made growth chamber equipped with a LED lighting system. Two specific light regimes were selected: broad-spectrum white (W) and red-blue light (R:B, 60:40, with emission peaks at 620 and 660 nm for red and emission peak at 460 nm for blue).The spectral composition of light regimes is reported in Figure 6 and was determined by a SpectraPen mini radiometer at 1nm resolution (Photon System Instruments, spol. S.r.o., Czech Republic).

All plants were grown in the same environmental conditions: PPFD of 350 µmol (photon) m^−2^ s^−1^ at the top of the canopy, 25/15 °C day/night temperature, 50/70% day/night relative humidity, and photoperiod of 12 h. Plants were watered to field capacity and fertilized every week with a complete nutritive solution (N:P:K, 20:20:20) (Poly-Feed GG, Haifa Italia, Bologna). We applied to soil at sowing time and weekly for three consecutive weeks, using a commercial biofertilizer (RadiNET, Micosat F^®®^, C.C.S. Aostas.r.l., Aosta, Italy) containing arbuscular mycorrhizal fungi (AMF) (*Glomus* genus, *Rhizophagus irregularis*), saprophytic fungi (*Pochonia chlamydosporia*, *Tricoderma* genus), and rhizosphere bacteria (*Bacillus* and *Streptomyces* genus). For each application, 0.6 g of biofertilizer was dissolved in 10 mL of deionized water. Five plants for each light regime (W and RB) were treated with biofertilizer (inoculated plants–I) and without (not-inoculated plants—NI) for a total of 10 plants for each light regime (5 plants for W-NI, 5 plants for RB-NI, 5 plants for W-I, and 5 plants for RB-I).

Plants were grown up to 100 days after sowing (DAS). Then, we evaluated the effect of light quality, PGPM application, and their interaction on biometrical and physiological parameters before the harvesting.

### 4.2. Biometrical Characteristics

The leaf number (n plant^−1^), the total leaf area, and the biomass determinations were evaluated on five plants per treatment. The digital image of every single leaf was analyzed with the Image J software (Image Analysis Software, Rasband, NIH, Bethesda, MD, USA). The sum of all leaf areas was used to determine the total leaf area per plant (cm^2^ plant^−1^). At harvest time, roots and shoots were disposed into the oven and dried at 75 °C until a constant dry weight was achieved. Then, the total biomass per plant and the ratio shoot/total biomass and root/total biomass were determined.

### 4.3. Photosynthetic Response Curves

Light response curves (LRCs) were performed using the LI-6400 (Li-Cor, Lincoln, NE, USA) integrated with LI-6400-40 leaf chamber fluorometer. LRCs were performed at 25 °C, 360 µmol (CO_2_) mol^−1^, and 50% air relative humidity (R.H.) by exposing fully expanded mature leaves at increasing light intensities (I) ranging from 0 to 1500 µmol (photons) m^−2^ s^−1^. For each light intensity used to build up the LRCs, the gas exchange parameters, namely net photosynthesis (*P*_N_), stomatal conductance (*g*_s_), transpiration (*T*_r_), and intercellular CO_2_ concentration (*C*_i_), were calculated by the software operating in Licor-6400, according to von Caemmerer and Farquhar [31]. The instantaneous water use efficiency (WUE_i_) was calculated as *P*_N_/*T*_r_ ratio, whereas the intercellular (*C*_i_) and the atmospheric (*C*_a_) CO_2_ concentrations were used to calculate the *C*_i_/*C*_a_ ratio.

To describe the *P*_N_/I curve, we used the three parameters for the exponential rise to the max equation:

*P*_N_ = *R*_d_ + *P*_Nmax_ (1 − e^−(Q_app_/*P*_Nmax_)PPFD^)

where *R*_d_ is the dark respiration, *P*_Nmax_ is the light-saturated net photosynthesis, and Q_app_ is the apparent light efficiency, representing the maximum quantum yield in the linear part of the curve.

The fluorescence parameters such as the quantum yield of linear electron transport (Φ_PSII_) [32], the quantum yield of regulated (Φ_NPQ_), and non-regulated (Φ_NO_) energy dissipation were calculated by the software operating in Licor-6400 for each light intensity according to Kramer et al. [33]. The maximum photochemical efficiency of photosystem II, *F*_v_/*F*_m_, was determined on 30 min dark-adapted leaves at the end of the LRCs.

CO_2_ response curves (CRCs) were also performed by exposing leaves to different CO_2_ concentrations, ranging from 50 to 1400 μmol mol^−1^, upon a saturation light of 700 μmol (photons) m^−2^s^−1^ as determined from LRC. *P*_N_ and the electron transport rate (*J*_f_) were calculated by the software operating in Licor-6400 for each CO_2_ step following Krall and Edwards [34]. For both LRCs and CRCs, we mainly analyzed two regions: the linear region (phase 1) and the saturation region (phase 2) of curves. For LRCs, phase 1 fell within the light-limited region, whereas phase 2 fell within the Rubisco-limited region. For CRCs, phase 1 fell within the Rubisco-limited zone, while phase 2 fell within the RuBP-limited zone.

Total chlorophyll concentration was measured by SPAD-502 portable chlorophyll meter (Minolta, Osaka, Japan) on the same leaves used for the LRC measurements.

### 4.4. Rubisco Quantification

Total protein extraction was performed on five leaves (one leaf per plant) per treatment, utilizing 0.3 g of fresh material for each sample according to the method reported in Wang et al. [35]. The extracts were quantified by the Bradford assay (BioRad Protein Assay Dye Reagent Concentrate; Bio-Rad Laboratories, Milan, Italy), determining the absorbance at 595 nm by a spectrophotometer (UV-VIS Cary 100; Agilent Technologies, Palo Alto, CA, USA). Bovine serum albumin (BSA) was used as the standard. SDS-PAGE (10%) was performed following the procedure of Vitale et al. [36], using Pro-liner 3-colour (Cyanagen Srl, Bologna, Italy) as a marker and Laemmli loading buffer to track the separation of proteins. Western blot analysis was carried out with a blocking solution (100 mM Tris-HCl pH 8.0, 150 mM NaCl, 0.1% Tween 20, 2.5% BSA) and primary antibodies (Agrisera, Vännäs, Sweden) to reveal Rubisco (anti-RbcL, rabbit polyclonal serum, 1:10,000 *v/v*, AS03037), and Actin protein (anti-ACT, rabbit polyclonal, 1:5000 *v/v*, AS132640) was utilized as a loading control. Anti-Rabbit IgG (H&L) and HRP conjugated (1:6000 *v/v*, AS09602) was used as a secondary antibody. The immune revelation was carried out with the kit for chemiluminescence (Westar supernova, Cyanagen Srl, Bologna, Italy) via ChemiDoc System (Bio-Rad). The Image J 1.45 program (Image Analysis Software, NIH, Bethesda, MD, USA) was utilized for the densitometric analysis to obtain quantitative information associated with the individual bands. Each Rubisco band was normalized to the corresponding actin band. Density values were expressed in arbitrary units and represented as bar diagrams showing pixel volumes of protein bands.

### 4.5. Statistical Analysis

All data were analyzed using SigmaPlot 12 software (Jandel Scientific, San Rafael, CA, USA). The two-way ANOVA was applied to assess the effect of the two different independent factors, i.e., light quality regimes (LQ), plant-growth-promoting microorganisms’ application (PGPM), and their possible interaction (LQ × PGPM) on biometrical characteristics, light and CO_2_ response curve-derived photosynthetic functional traits, chlorophyll, and Rubisco amount. The normality was verified with the Shapiro–Wilk test, while the Student–Newman–Keuls (SNK) test was applied for all pairwise multiple comparisons with a significance level of *p* ≤ 0.05. For a significant interaction LQ × PGPM, we used the one-way ANOVA and SNK coefficient for multiple comparisons.

## 5. Conclusions

Based on our results, we conclude that the PGPM cocktail used in this study positively influenced the photosynthetic performance of spinach plants. These benefits were obtained by improving in inoculated plants the efficiency of photosystem II, enhancing both the electron transport rate and PSII photochemical efficiency, especially in phase 2 of LRCs and CRCs. The beneficial effects of PGPM were reduced under dichromatic RB light regime, likely due to the lack of critical wavelengths useful for photosynthesis other than red and blue. Under RB, the costs for plant–microbes symbiosis were likely greater than under full light spectrum, leading to a small increase in photosynthesis. We conclude that plant-growth-promoting microbes used to enhance plant productivity and yield quality alter the photosynthetic response to light quality. This issue must be considered when PGPM are used to improve plant primary production in controlled environments under artificial lighting.

## Figures and Tables

**Table 1:** Analysis of variance and comparison of means for leaf number, total leaf area, shoot/total biomass, root/total biomass of plants in response to light quality (LQ), plant-growth-promoting microbes (PGPM), and their interaction (LQ × PGPM). Different letters in each column indicate significant differences according to Student–Newman–Keuls (SNK) test (*p* ≤ 0.05). NS—not significant; ** *p* ≤ 0.01.

	Leaf Number	Total Leaf Area	S/B	R/B
**LQ**				
W	14 ± 0.9 a	60 ± 3.7 a	0.74 ± 0.03 a	0.28 ± 0.03 b
RB	13 ± 0.9 a	49 ± 2.9 b	0.62 ± 0.03 b	0.50 ± 0.06 a
**PGPM**				
NI	13 ± 0.6 a	55 ± 2.9 a	0.73 ± 0.04 a	0.36 ± 0.07 a
I	14 ± 1.2 a	53 ± 5.2 a	0.63 ± 0.012 b	0.42 ± 0.03 a
**Condition**				
W-NI	13 ± 0.4 a	58 ± 3.9 a	0.82 ± 0.03 a	0.22 ± 0.05 b
RB-NI	13 ± 0.9 a	52 ± 3.6bc	0.64 ± 0.05 b	0.49 ± 0.12 a
W-I	15 ± 1.3 a	62 ± 0.9 a	0.66 ± 0.01 b	0.34 ± 0.01 b
RB-I	13 ± 1.1 a	45 ± 3.4 c	0.60 ± 0.01 b	0.51 ± 0.02 a
**Significance**				
LQ	NS	**	**	**
PGPM	NS	NS	**	NS
LQ x PGPM	NS	NS	NS	NS

Leaf number: number of leaves per plant (n plant^−1^); Total leaf area: total leaf area per plant (cm^2^ plant^−1^); S/B: dry shoot biomass/total dry biomass per plant; R/B: dry root biomass/total dry biomass per plant. Different letters denote significant differences. Data are means± standard error (n = 5).

**Table 2:** Analysis of variance and comparison of means for the light response curve (LRCs)-derived photosynthetic functional traits *P*_N(LRC)_, g_s_, *C*_i_/*C*_a_, WUE_i_, Φ_PSII_, Φ_NPQ_, Φ_NO_ in response to light quality (LQ), plant-growth promoting microbes (PGPM), and their interaction (LQ × PGPM). Different letters in each column indicate significant differences according to Student–Newman–Keuls (SNK) test (*p* ≤ 0.05). NS—not significant; * *p* ≤ 0.05; ** *p* ≤ 0.01; *** *p* ≤ 0.001.

Light Response Curve							
	*P*_N (LRC)_	*g*_s_	*C*_i_/*C*_a_	WUE_i_	Φ_PSII_	Φ_NPQ_	Φ_NO_
**Light**							
W	a	b	a	a	a	a	a
RB	a	a	a	a	a	a	b
**PGPM**							
NI	b	b	a	a	a	a	a
I	a	a	a	a	a	a	b
**Significance**							
LQ	NS	***	NS	NS	NS	NS	**
PGPM	*	***	NS	NS	NS	NS	**
LQ × PGPM	NS	***	NS	NS	NS	NS	**
**Phase 1**							
	***P*_N (LRC)_**	***g*_s_**	***C*_i_/*C*_a_**	**WUE_i_**	**Φ_PSII_**	**Φ_NPQ_**	**Φ_NO_**
**Light**							
W	a	b	a	a	a	a	a
RB	a	a	a	a	a	a	b
**PGPM**							
NI	a	b	b	a	a	a	a
I	a	a	a	b	a	a	a
**Combination**							
W-NI	c	c	b	a	c	ab	a
W-I	b	a	a	bc	ab	ab	a
RB-NI	ab	b	b	b	bc	b	b
RB-I	a	a	a	c	a	a	b
**Significance**							
LQ	NS	***	NS	NS	NS	NS	**
PGPM	NS	***	**	**	NS	NS	NS
LQ × PGPM	*	*	*	*	*	*	NS
**Phase 2**							
	***P*_N (LRC)_**	***g*_s_**	***C*_i_/*C*_a_**	**WUE_i_**	**Φ_PSII_**	**Φ_NPQ_**	**Φ_NO_**
**Light**							
W	b	b	a	a	a	a	a
RB	a	a	a	a	a	a	b
**PGPM**							
NI	b	b	b	a	b	a	a
I	a	a	a	b	a	b	b
**Combination**							
W-NI	d	d	b	a	c	ab	a
W-I	c	b	a	bc	ab	ab	b
RB-NI	b	c	b	b	b	b	b
RB-I	a	a	a	c	a	a	b
**Significance**							
LQ	***	***	NS	NS	NS	NS	*
PGPM	***	***	***	***	*	*	*
LQ × PGPM	*	***	NS	NS	NS	NS	*

*P*_N(LRC)_: net photosynthetic rate; *g*_s_: stomatal conductance; *C*_i_/*C*_a_: internal to atmospheric CO_2_ concentration ratio; WUE_i_: instantaneous water use efficiency; Φ_PSII_: effective quantum yield of PSII; Φ_NPQ_: regulated energy dissipation of PSII; Φ_NO_: not-regulated energy dissipation of PSII. Different letters denote significant differences. LRC comparation data: n = 200; data for phase 1 comparation: n = 80; data for phase 2 comparation: n = 80.

**Table 3:** Analysis of variance and comparison of means for the CO_2_ response curve (CRCs)-derived photosynthetic functional traits *P*_N_ (CRC) and *J*_f_ in response to light quality (LQ), plant-growth promoting microbes (PGPM), and their interaction (LQ × PGPM). Different letters in each column indicate significant differences according to Student–Newman–Keuls (SNK) test (*p* ≤ 0.05). NS—not significant; * *p* ≤ 0.05; ** *p* ≤ 0.01; *** *p* ≤ 0.001.

CO_2_ Response Curve		
	*P*_N (CRC)_	*J*_f_
**Light**		
W	a	b
RB	a	a
**PGPM**		
NI	a	b
I	a	a
**Significance**		
LQ	NS	*
PGPM	NS	**
LQ × PGPM	NS	*
	**Phase 1**	
	***P*_N (CRC)_**	***J*_f_**
**Light**		
W	a	b
RB	a	a
**PGPM**		
NI	b	b
I	a	a
**Combination**		
W-NI	c	c
W-I	b	b
RB-NI	ab	b
RB-I	a	a
**Significance**		
LQ	NS	*
PGPM	*	***
LQ × PGPM	*	*
	**Phase 2**	
	***P*_N (CRC)_**	***J*_f_**
**Light**		
W	a	a
RB	a	a
**PGPM**		
NI	b	b
I	a	a
**Combination**		
W-NI	c	c
W-I	a	ab
RB-NI	b	bc
RB-I	a	a
**Significance**		
LQ	NS	NS
PGPM	*	***
LQ × PGPM	NS	NS

*P*_N (CRC)_: net photosynthetic rate; *J*_f_: electron transport rate. Different letters denote significant differences. CRC comparation data: n = 220; data for phase 1 comparation: n = 120; data for phase 2 comparation: n = 60.

**Table 4:** Analysis of variance and comparison of means for SPAD, photosynthetic functional traits plants in response to light quality (LQ), plant-growth-promoting microbes (PGPM), and their interaction (LQ × PGPM). Different letters in each column indicate significant differences according to Student–Newman–Keuls (SNK) test (*p* ≤ 0.05). NS—not significant; * *p* ≤ 0.05; *** *p* ≤ 0.001.

	SPAD	*F*_v_/*F*_m_	*R*_d_	*P*_Nmax_	Q_pp_
**LQ**					
W	63 ± 1.2 a	0.772 ± 0.004 a	−2.11 ± 0.05 a	13.2 ± 0.64 b	0.089 ± 0.002 b
RB	60 ± 0.9 a	0.774 ± 0.005 a	−2.34 ± 0.05 b	15.3 ± 0.49 a	0.098 ± 0.001 a
**PGPM**					
NI	63 ± 0.8 a	0.770 ± 0.003 a	−2.27 ± 0.07 a	12.8 ± 0.47 b	0.090 ± 0.003 b
I	61 ± 1.3 a	0.777 ± 0.004a	−2.18 ± 0.12 a	15.7 ± 0.46 a	0.096 ± 0.002 a
**Condition**					
W-NI	64 ± 1.1 a	0.766 ± 0.001 a	−2.32 ± 0.14 ab	11.4 ± 0.16 d	0.084 ± 0.002 b
RB-NI	62 ± 1.3 a	0.772 ± 0.006 a	−2.22 ± 0.06 ab	14.1 ± 0.26 c	0.097 ± 0.002 a
W-I	63 ± 2.2 a	0.77 ± 0.006 a	−1.90 ± 0.15 a	14.9 ± 0.20 b	0.093 ± 0.003 a
RB-I	59 ± 1.1 a	0.77 ± 0.006 a	−2.46 ± 0.02 b	16.6 ± 0.52 a	0.099 ± 0.001 a
**Significance**					
LQ	NS	NS	*	***	*
PGPM	NS	NS	NS	***	***
LQ × PGPM	NS	NS	*	*	*

SPAD: Soil Plant Analysis Development chlorophyll index; *F*_v_/*F*_m_: maximal photochemical efficiency of photosystem II; *R*_d_: dark respiration; *P*_Nmax_: light-saturated net photosynthesis; Q_pp_: apparent light efficiency. Photosynthesis data derived from LRC. Data are means ± standard error (n = 5). Different letters denote significant differences.

## Data Availability

The data presented in this study are available on request from the corresponding author.
